# Laparoscopic Removal of a Prostatic Utricle in a 15-Month-Old Boy Under Cystoscopic Guidance: A Case Report and Review of the Literature

**DOI:** 10.7759/cureus.71826

**Published:** 2024-10-19

**Authors:** Elisavet Kanna, Zoi Lamprinou, Theodoros Spinos, Rodanthi Sfakiotaki, Ioannis Skondras

**Affiliations:** 1 2nd Pediatric Surgery Department, Panagiotis & Aglaia Kyriakou Childrens' Hospital, Athens, GRC; 2 Urology, General University Hospital of Patras, Patras, GRC; 3 Radiology Department, Panagiotis & Aglaia Kyriakou Childrens' Hospital, Athens, GRC

**Keywords:** children, enlarged prostatic utricle, laparoscopy, mullerian duct remnants, pmds

## Abstract

The prostatic utricle, also known as the utricle of the prostate or Müllerian duct cyst, is a small blind-ended tubular structure found within the prostate gland of some males. In this work, we report the case of a 15-month-old boy, with a history of recurrent epididymitis, presenting with new-onset left scrotal swelling and pain. On cystourethrogram, the patient had an enlarged prostatic utricle (EPU). A successful minimally invasive (laparoscopic) approach for the total excision of the prostatic utricle cyst was performed, while injury of the vas deferens was avoided. Identification and secure dissection of the utricle remnant were aided by the presence of a ureteric stent inside the utricle orifice. The patient had an uneventful postoperative course and remains asymptomatic up to now.

## Introduction

The prostatic utricle, a structure found within the prostate gland of males, is a vestigial remnant of the Müllerian ducts. These ducts are critical embryonic structures that typically develop into the female reproductive tract components, such as the uterus, fallopian tubes, and upper vagina [1,2]. However, in males, the Müllerian ducts usually regress during fetal development [1].

In some instances, a small portion of the Müllerian ducts may persist and give rise to the prostatic utricle. This structure is characterized by its tubular shape and is lined with glandular epithelium. It may contain fluid or mucus within its lumen.

While the prostatic utricle is often asymptomatic, there are rare cases where it can lead to complications. Enlargement or infection of the prostatic utricle may occur, resulting in symptoms such as urinary difficulties or sexual dysfunction. In such cases, surgical removal or drainage of the prostatic utricle is the preferred treatment modality.

The primary goals of surgical intervention are to alleviate symptoms, preserve fertility, and prevent the development of neoplastic changes within the prostatic utricle [3]. However, the management of asymptomatic cases remains controversial, and decisions regarding intervention should be made on a case-by-case basis, considering factors such as patient preferences and potential risks associated with surgery.

## Case presentation

A 15-month-old boy presented to the emergency department of our hospital with left scrotal swelling and pain, while a history of recurrent urinary tract infections was also reported. He had no other comorbidities and his external genitalia were normal. He was born full-term, and no anomalies were detected or suspected during routine prenatal ultrasound scans.

**Figure 1:**
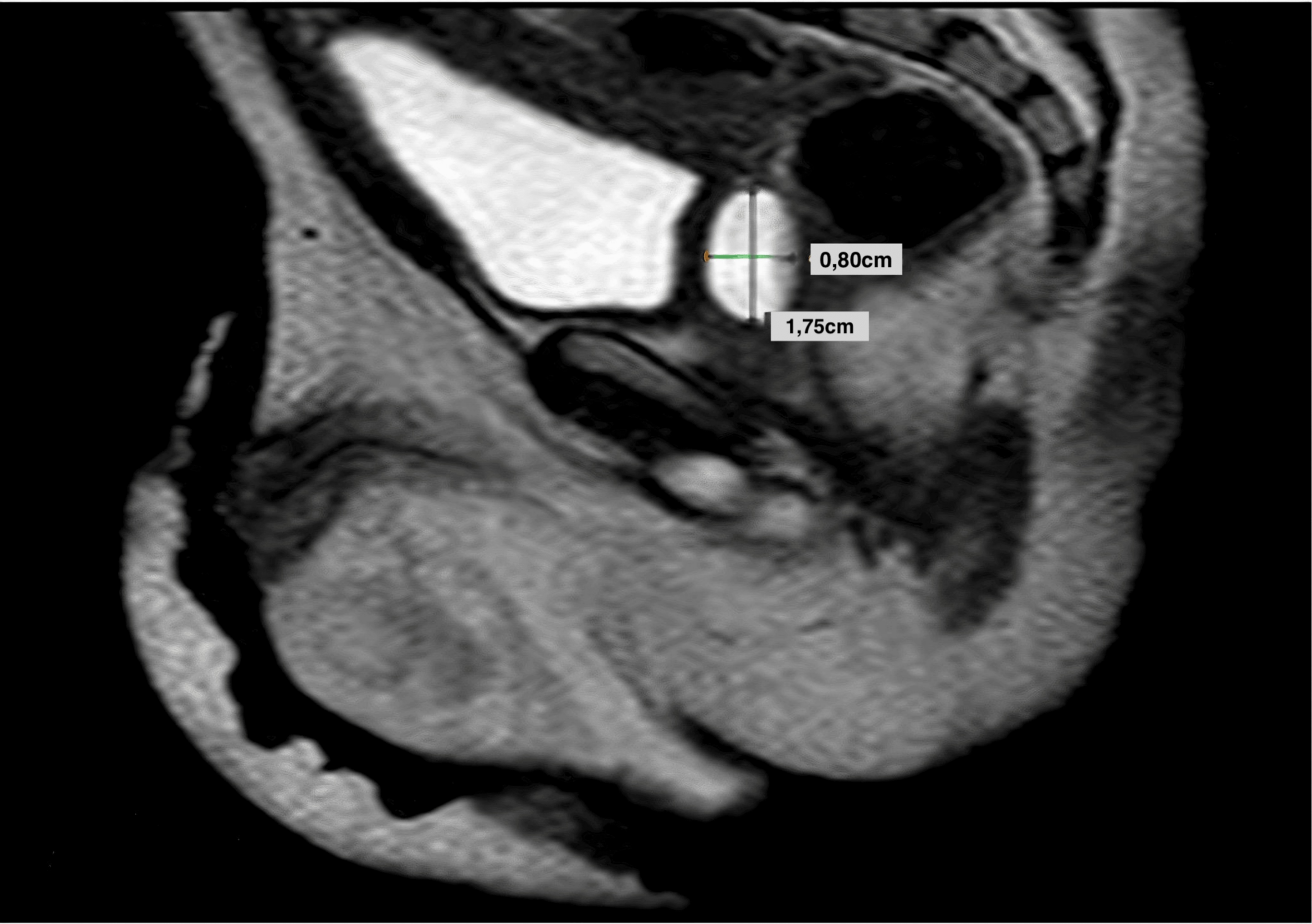
Magnetic resonance imaging prior to surgery shows the anatomical definition of the prostatic utricle

The patient continued to experience episodes of epididymitis, despite treatment with multiple antibiotics. Urine cultures revealed *Pseudomonas aeruginosa *which was sensitive to amikacin. As a result, we decided to perform a micturating cystourethrogram. The cystourethrogram demonstrated a cystic structure lying behind the bladder, which was filled on micturition and was interpreted as an enlarged prostatic utricle (EPU). An ultrasound scan of the upper and lower abdomen showed no dilatations along the urinary tract, excluding hydronephrosis and hydroureter. In order to obtain a better anatomical understanding of his internal genitalia and plan the surgery, the patient also underwent magnetic resonance imaging (MRI) of his upper and lower abdomen (Figure 1). We decided to proceed with total resection of the utricle cyst.

**Figure 2:**
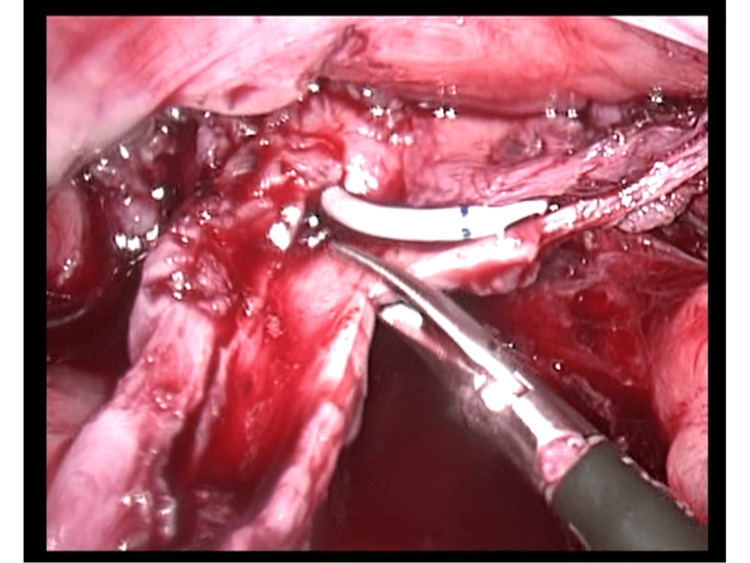
Dissection of the prostatic utricle with the ureteric stent as a guide

Under general anesthesia, a laparoscopic excision of the prostatic utricle cyst was performed. The patient was placed in lithotomy position, and a cystourethroscopy, which preceded the laparoscopic excision, confirmed the diagnosis and identified the utricle’s orifice in the verumontanum. The cystoscope was then passed into the utricular cyst and a 3.7 FR ureteric stent was left in position so as to aid the subsequent laparoscopic identification. A 10 mm trocar was inserted into the umbilical position, and a pneumoperitoneum was created. Two other 3 mm trocars were inserted, under direct vision, in the left and in the right iliac fossa, respectively. The pneumoperitoneum pressure was set at 8-10 mmHg throughout the procedure with the aid of the AirSeal System. After bladder suspension, the cyst identified with the help of the ureteric stent followed by dissection and isolation from the surrounding tissues in an attempt to minimize any damage to the adjacent structures. Emphasis was given into avoiding injury of the vas deferens. Right spermatic cord was densely adherent to the wall of the cyst . For better dissection, a traction loop suture was place around the cord with the help of a monofilament suture 3/0 passed through the abdominal wall. Once the prostatic utricle was adequately dissected, it was carefully excised from the prostate gland having the ureteric stent as a guide so to avoid urethral damage. The posterior wall was sutured with interrupted 4/O 17 mm (Figure 2). The specimen was removed together with one of the 3 mm trocar at the end of the operation. The total operative time was 176 minutes. Postoperative hemoglobin was 9.7 g/dl (the preoperative value was 10.1 g/dl). The intraoperative and postoperative course was uneventful, drain was removed on day 2, and the patient was discharged from the hospital on postoperative day 3. The Foley catheter was removed day 7 in the outpatient department. The pathologist’s report confirmed that the histology was compatible with prostatic utricle without the presence of the vas deferens.

**Figure 3:**
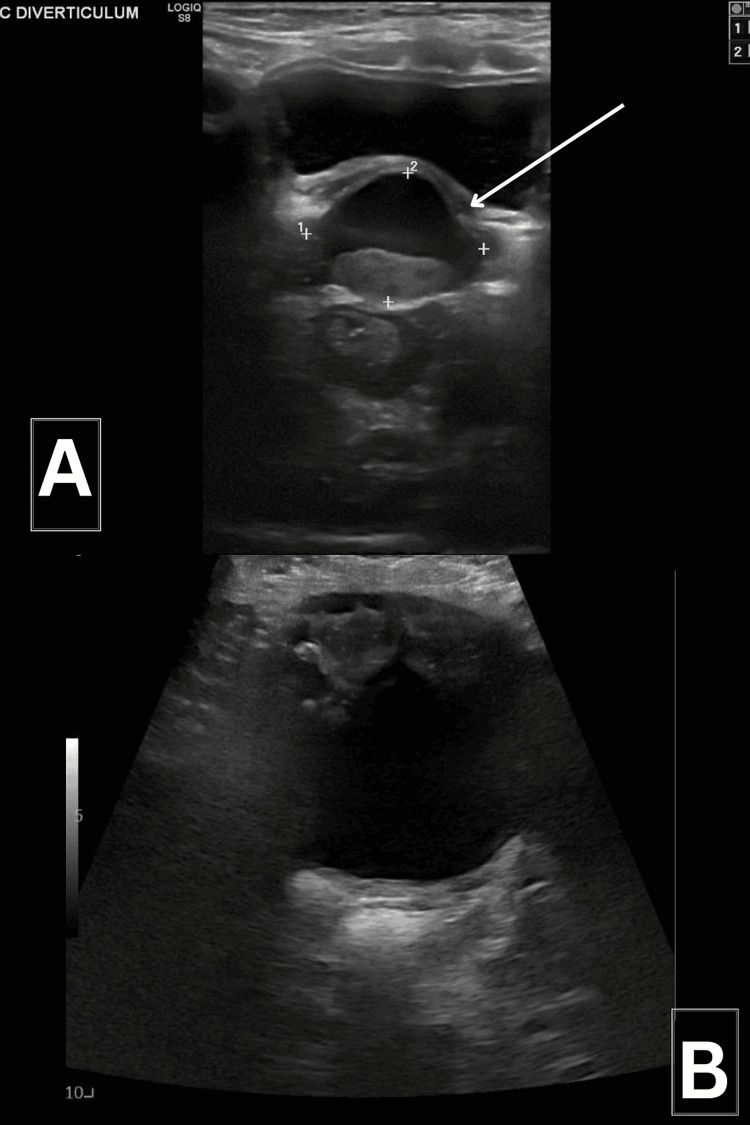
Bladder ultrasound (A) prior to surgical excision (B) postoperatively after the successful laparoscopic excision of the prostatic utricle cyst

The patient remains asymptomatic up to now for more than 24 months of follow-up with no residual or recurrent symptoms (Figure 3). 

## Discussion

The Müllerian ducts, the embryonic structures that give rise to the female reproductive organs (uterus, fallopian tubes, and upper vagina), regress and vanish throughout normal male fetal development [1]. Nevertheless, persistent Müllerian duct syndrome (PMDS), a disorder of sexual development, can result from the Müllerian ducts persisting, partially or entirely in certain situations. 

An autosomal recessive mode of inheritance characterizes PMDS, which is an infrequent medical condition involving a form of 46,XY disorder of sex development (DSD)[4]. It is caused by mutations in genes that are involved in the function and development of the male reproductive system, such as the anti-Müllerian hormone (AMH) gene. A hormonal imbalance in AMH and testosterone secretion or the AMH responsiveness of target tissues during a short critical period at the end of the undifferentiated stage (gestational weeks 9 to 10) can describe the condition. Undescended testis and posterior hypospadias are often associated with this syndrome, due to the above-mentioned hormonal imbalance [3].

One of the manifestations of PMDS is the presence of a prostatic utricle, which represents a small blind-ended tubular-shaped structure, communicating with the prostatic urethra. Many utricles are asymptomatic. An EPU may present as recurrent urinary tract irritative symptoms, voiding dysfunctions, or urinary incontinence due to secondary trapping of urine in the diverticulum [5]. EPU is further divided into grades 0-III, based on the findings of the cystourethrogram. Grade 0 represents a utricle which is confined to the verumontanum, while a grade I utricle lies below the bladder neck. Grade II stands for a utricle that extends over the bladder neck, and finally, a grade III utricle is associated with an opening distally to the external sphincter, in the bulbous urethra [6,7]. 

Treatment is aimed at relieving symptoms, preserving fertility, and preventing neoplastic degeneration [1]. Surgical excision of a prostatic utricle may be considered in certain cases, particularly if it is causing symptoms or complications. Regarding the conventional open surgery, several surgical approaches have been proposed for the treatment of EPU, including a perineal, retropubic, transvesical, transperitoneal, transanorectal, and perirectal or pararectal ones. All of these procedures tend to be technically complex and typically involve extended hospital stays. Furthermore, they carry the extra risk of complications, such as infections, incontinence, and impotence [6].

However, laparoscopy can potentially overcome these obstacles, providing an optimal view due to imaging magnification. It allows fine dissection of the EPU and excellent preparation of all adjacent anatomical structures, with a minimal access into the peritoneal cavity. Traction suture for more sharp spermatic cord dissection can be really helpful. It is also potentially associated with reduced morbidity from iatrogenic injury and a low incidence of postoperative adhesions. In our case, undoubtedly the presence of the ureteric catheter or the cystoscope within the utricular remnant significantly aided the dissection of the EPU by acting as a "guiding light"[2]. There have been described a few cases of a minimally invasive approach (laparoscopic or robotic-assisted) for the removal of EPU [2,3,6,8-10].

## Conclusions

It is uncommon to discover patients with prostatic utricles without hypospadias, cryptorchidism, or DSD, like in our case. The diagnosis is set based on the combination of clinical symptoms, physical examination, and imaging findings. Small asymptomatic prostatic utricles which are accidentally diagnosed can typically be closely monitored. Surgery is indicated for the treatment of big symptomatic and complicated utricles. Regarding conventional open surgery, many approaches have been described. Our case report, along with other reports found in the literature, show that laparoscopic excision is a feasible, efficient, and safe approach for the surgical treatment of symptomatic utricle cysts in childhood when performed in centers with experience in laparoscopic procedures.
